# Supplementary material for: Ultrasensitive and multiplexed protein imaging with clickable and cleavable fluorophores
Source: bioRxiv. 2023 Oct 23:2023.10.20.563323. Preprint. [Version 1] doi: 10.1101/2023.10.20.563323 (PMC10634699; doi:10.1101/2023.10.20.563323)
Supplement: Supplement 1 [file media-1.pdf]

Supporting Information: Ultrasensitive and multiplexed protein imaging with clickable and cleavable fluorophores

Thai Pham, Yi Chen, Joshua Labaer and Jia Guo*

Biodesign Institute & School of Molecular Sciences, Arizona State University, Tempe, Arizona 85287, United States

* Correspondence: jiaguo@asu.edu; Tel.: ((+1) 480-727-2096)

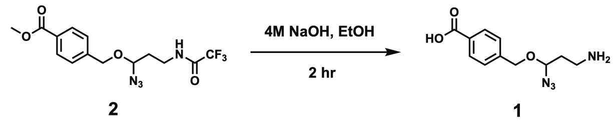

Figure S1. Synthetic Scheme of azido cleavable linker

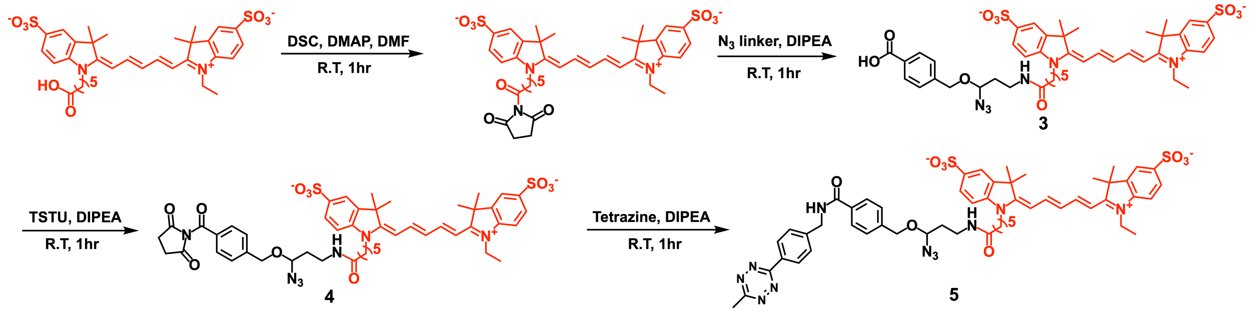

Figure S2. Synthetic Scheme of Tetrazine-N3-Cy5

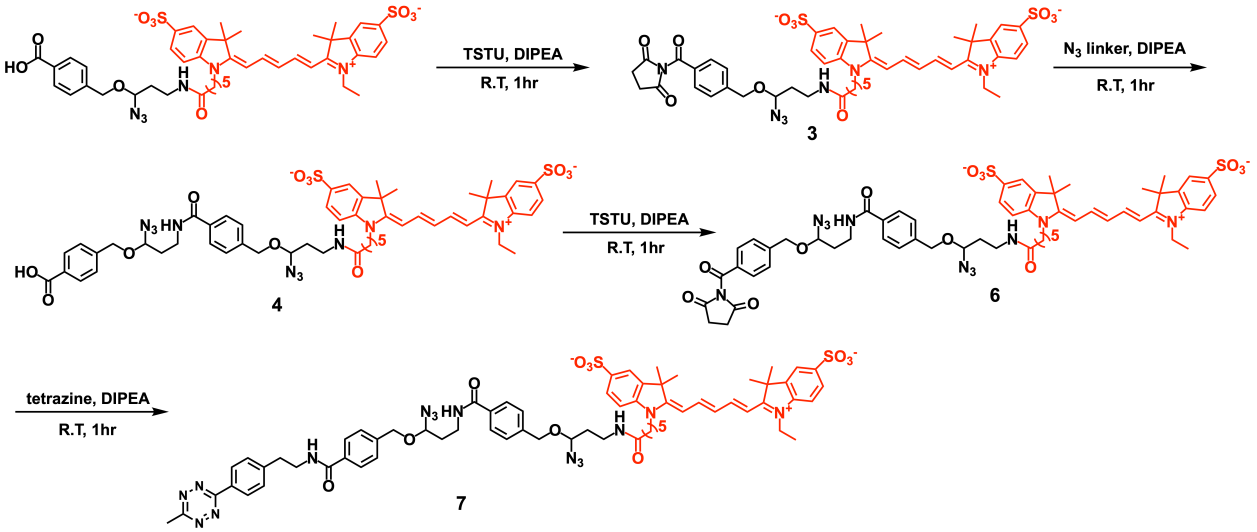

Figure S3. Synthetic Scheme of Tetrazine-N_3-N_3-Cy5

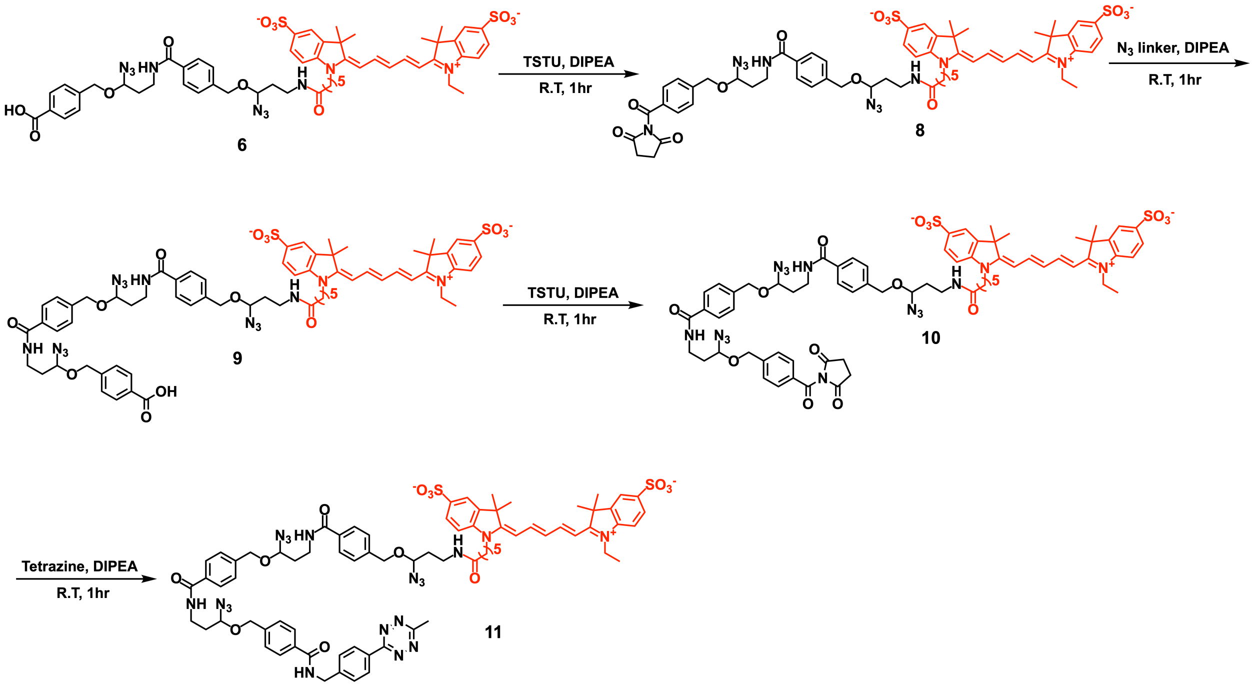

Figure S4. Synthetic Scheme of Tetrazine-N_3-N_3-N_3-Cy5

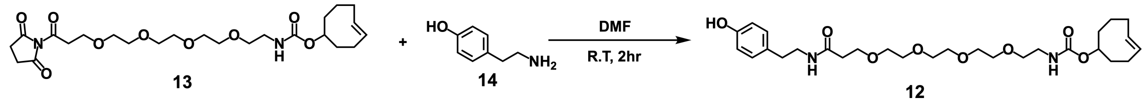

Figure S5. Synthetic Scheme of Tyramide-TCO

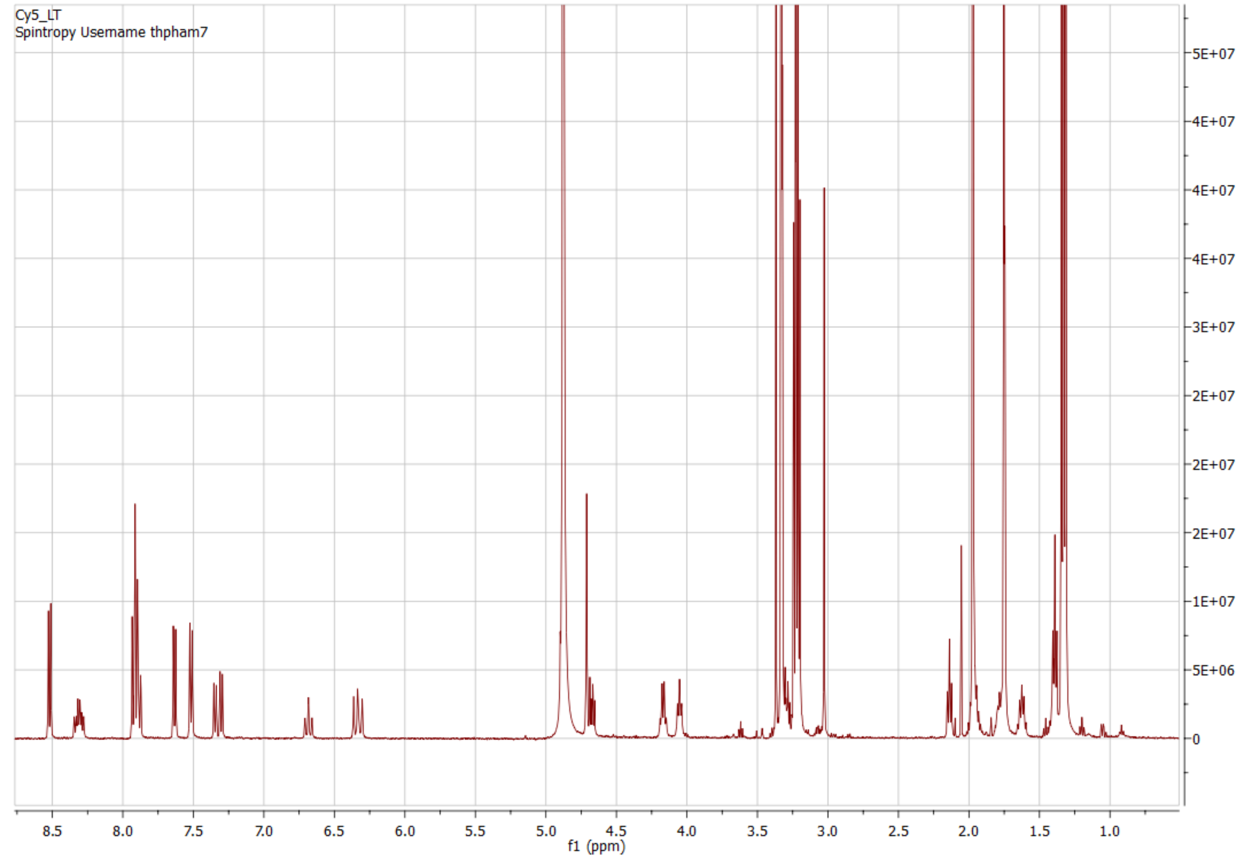

Figure S6. 500MHz ^{1}H NMR spectra of Tetrazine-N_3-Cy5 in CD_3OD

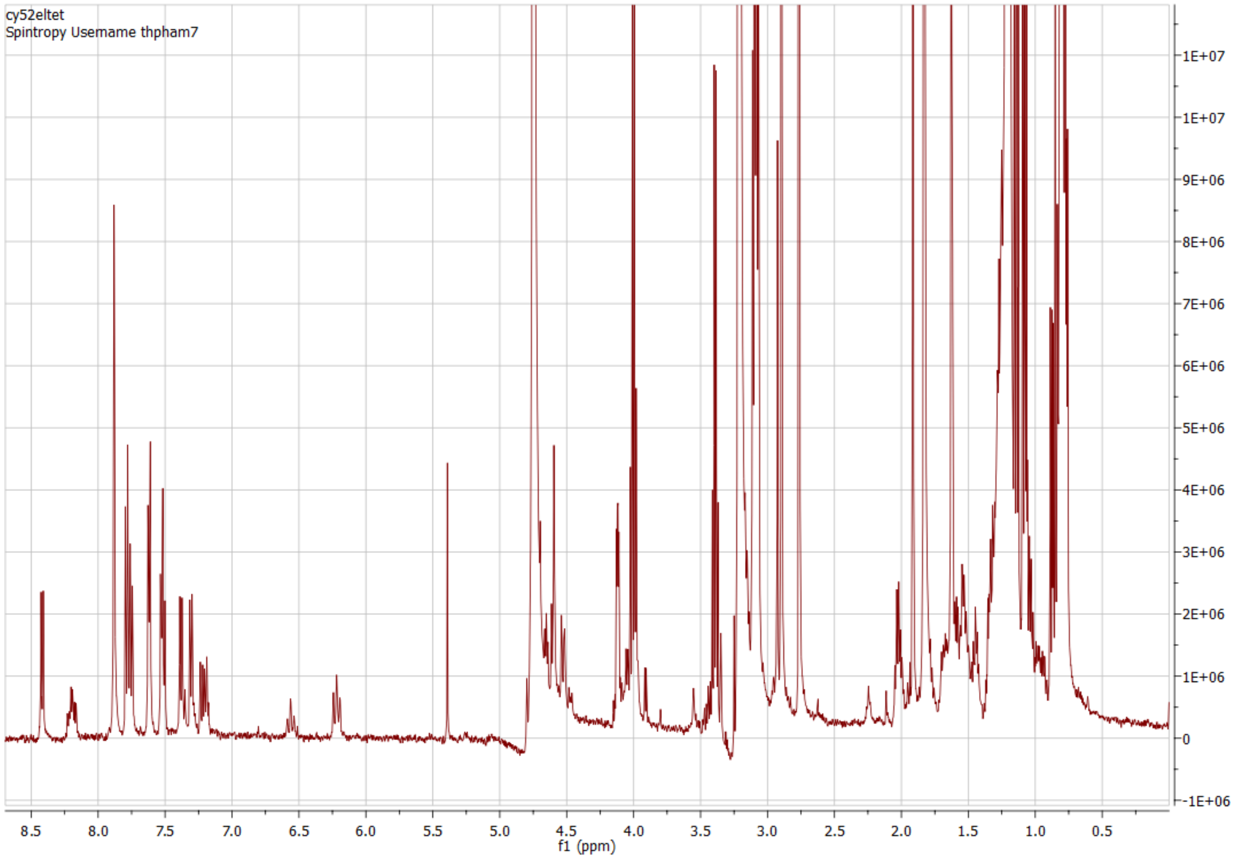

Figure S7. 500MHz ^{1}H NMR spectra of Tetrazine-N_3-N_3-Cy5 in CD_3OD

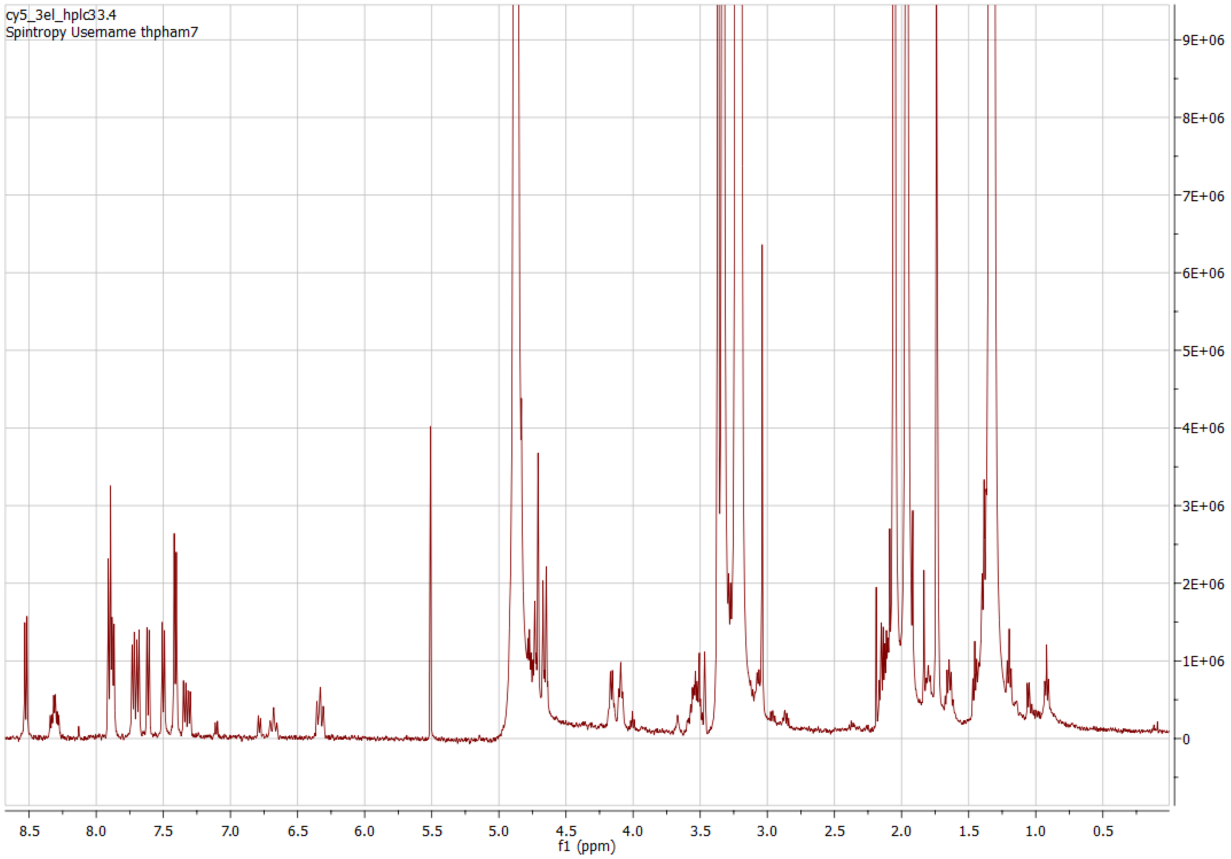

Figure S8. 500MHz ^{1}H NMR spectra of Tetrazine-N_3-N_3-N_3-Cy5 in CD_3OD

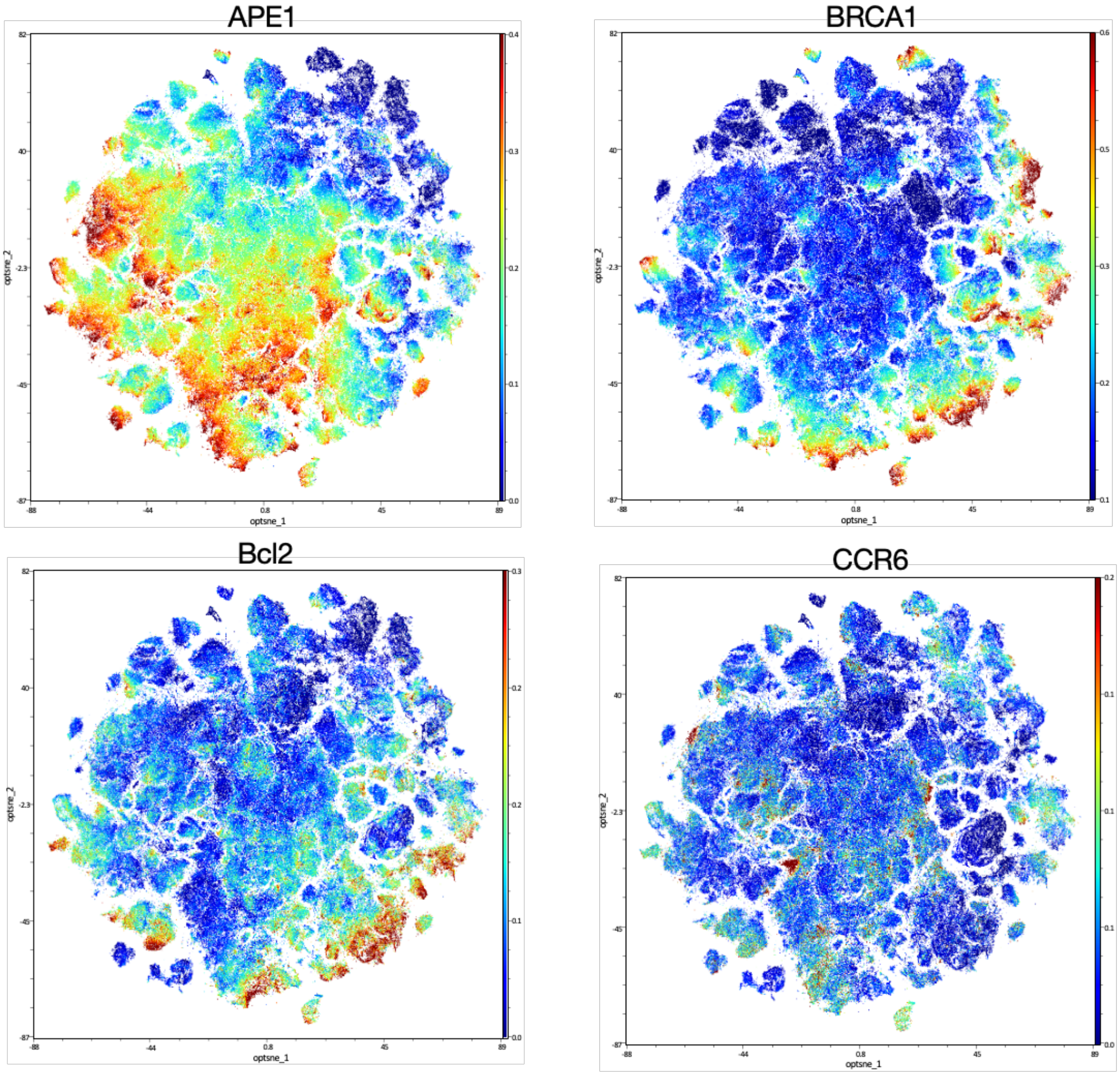
APE1
BRCA1
Bcl2
CCR6
optsne_1
optsne_2

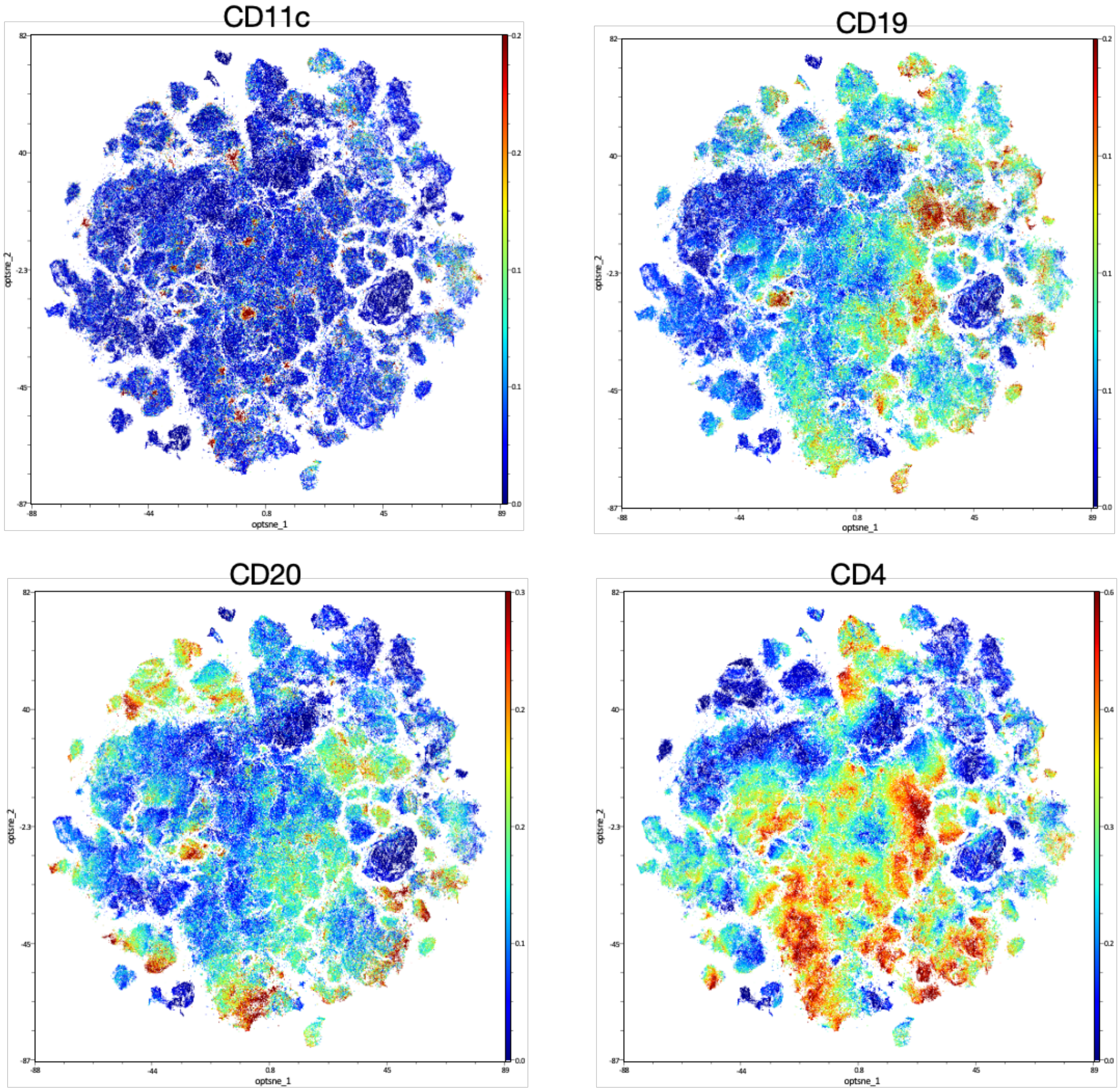
CD11c
CD19
CD20
CD4
optsne_1
optsne_2

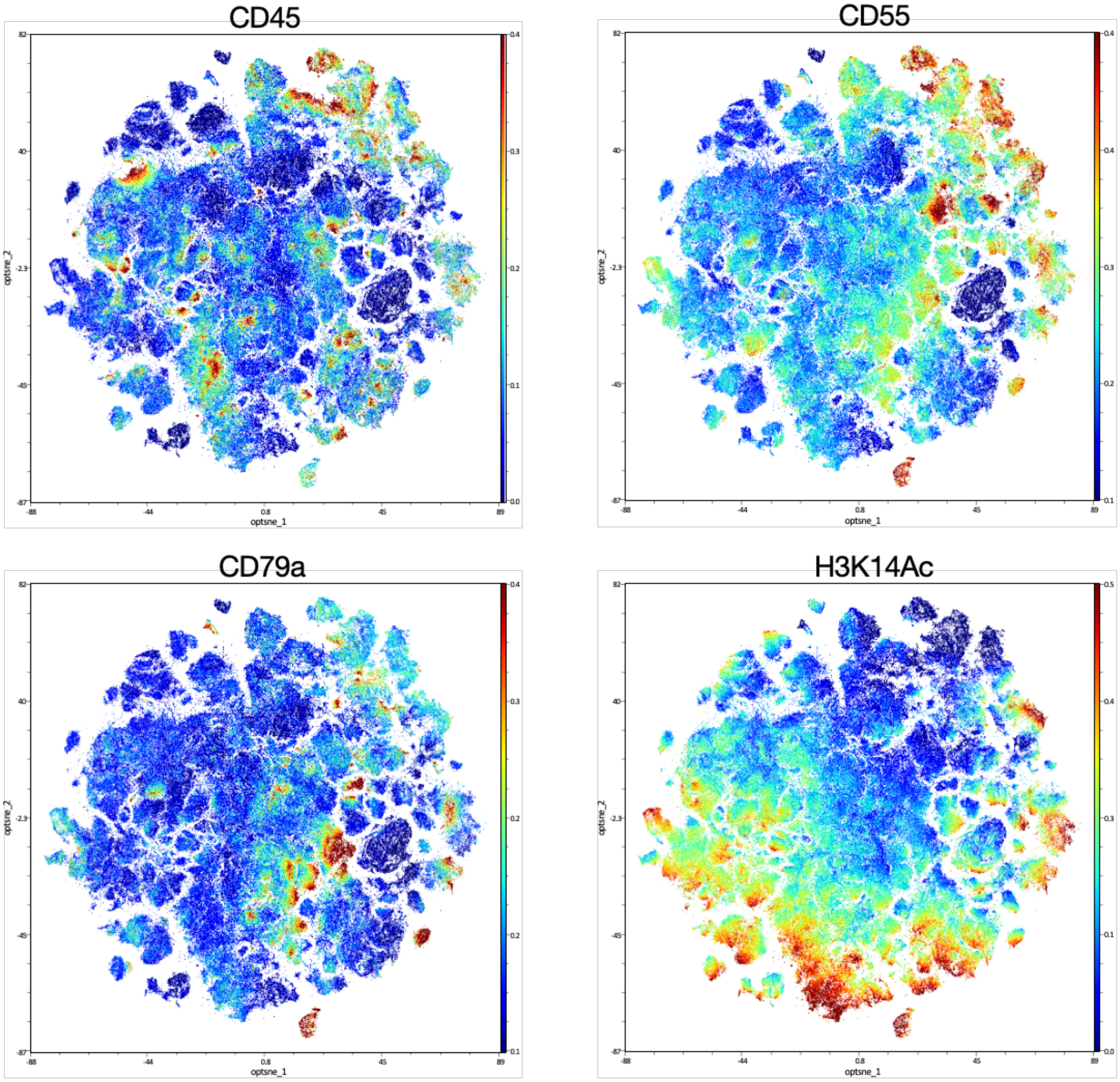

CD45
CD55
CD79a
H3K14Ac
optsne_1
optsne_2

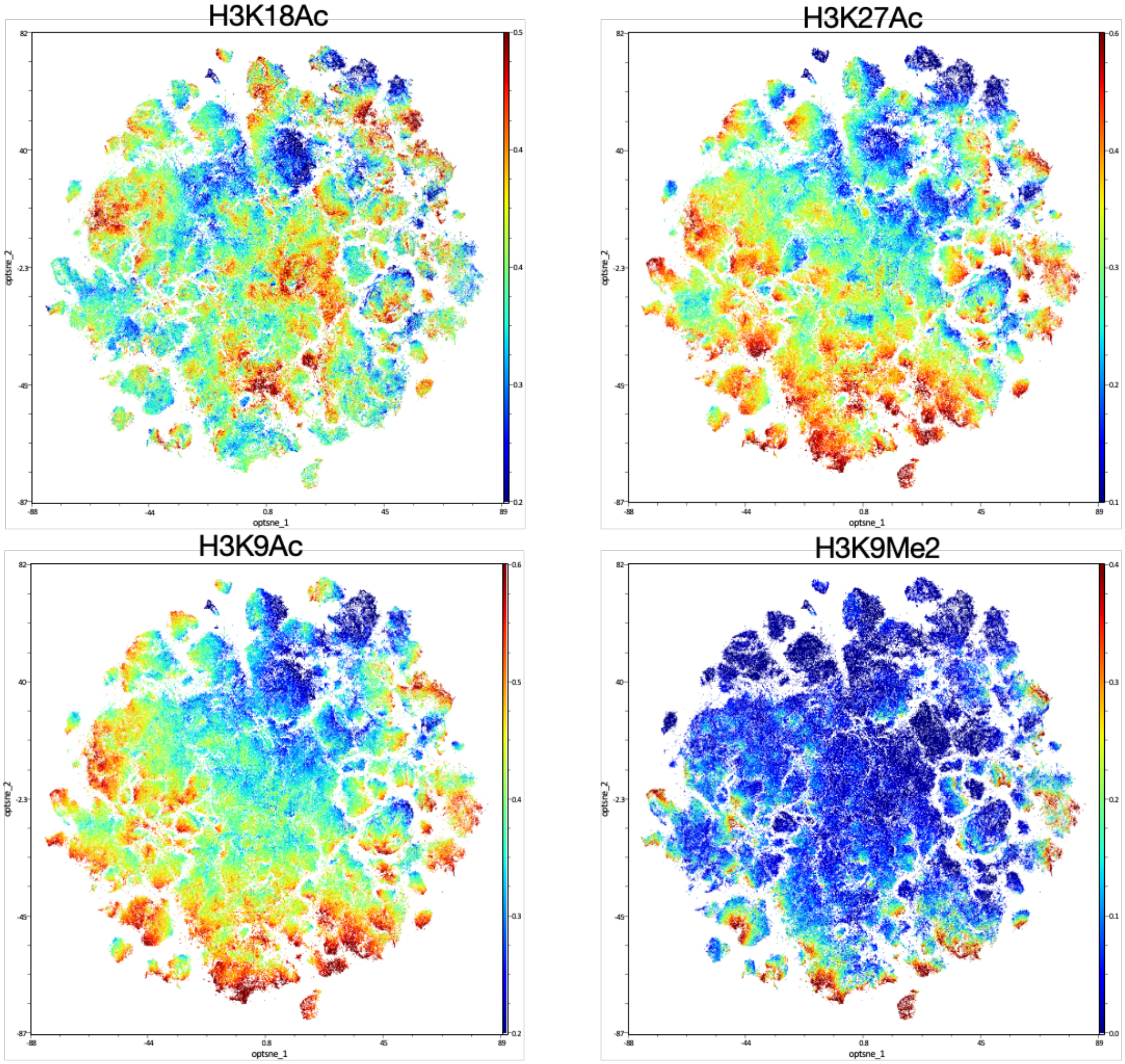
H3K18Ac
H3K27Ac
H3K9Ac
H3K9Me2
optsne_1
optsne_2

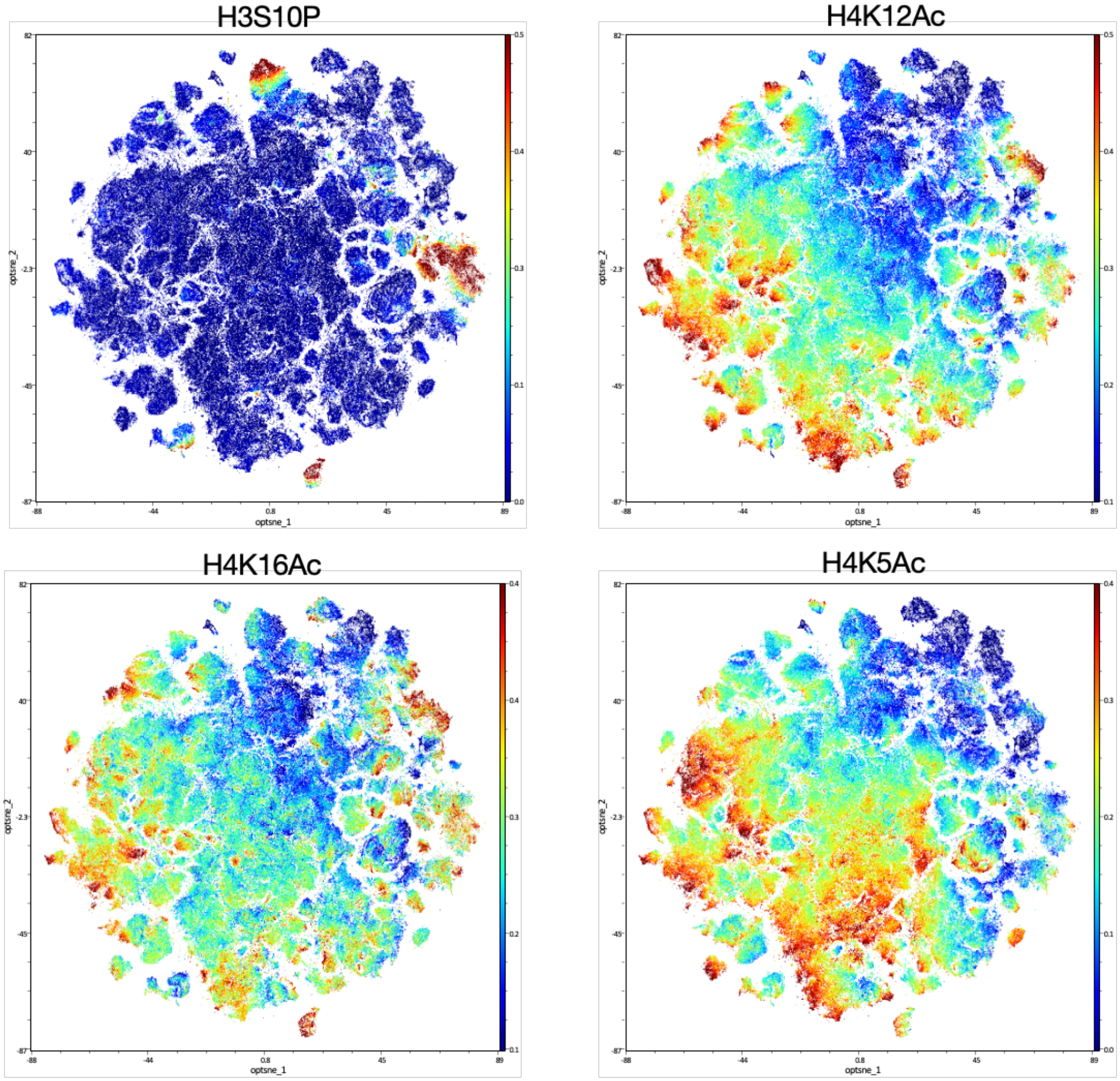
H3S10P
H4K12Ac
H4K16Ac
H4K5Ac
optsne_1
optsne_2

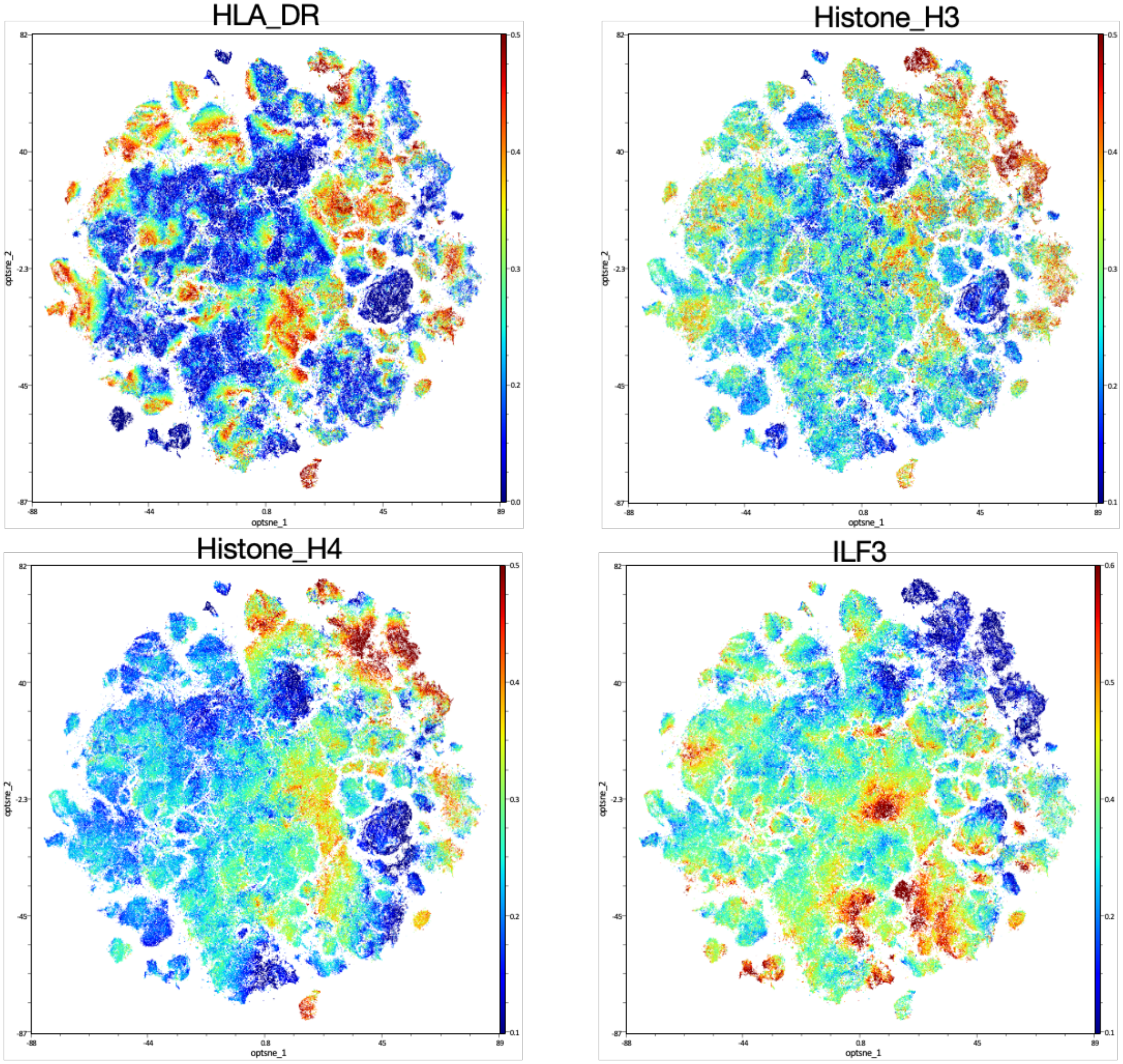
HLA_DR
Histone_H3
Histone_H4
ILF3
optsne_1
optsne_2

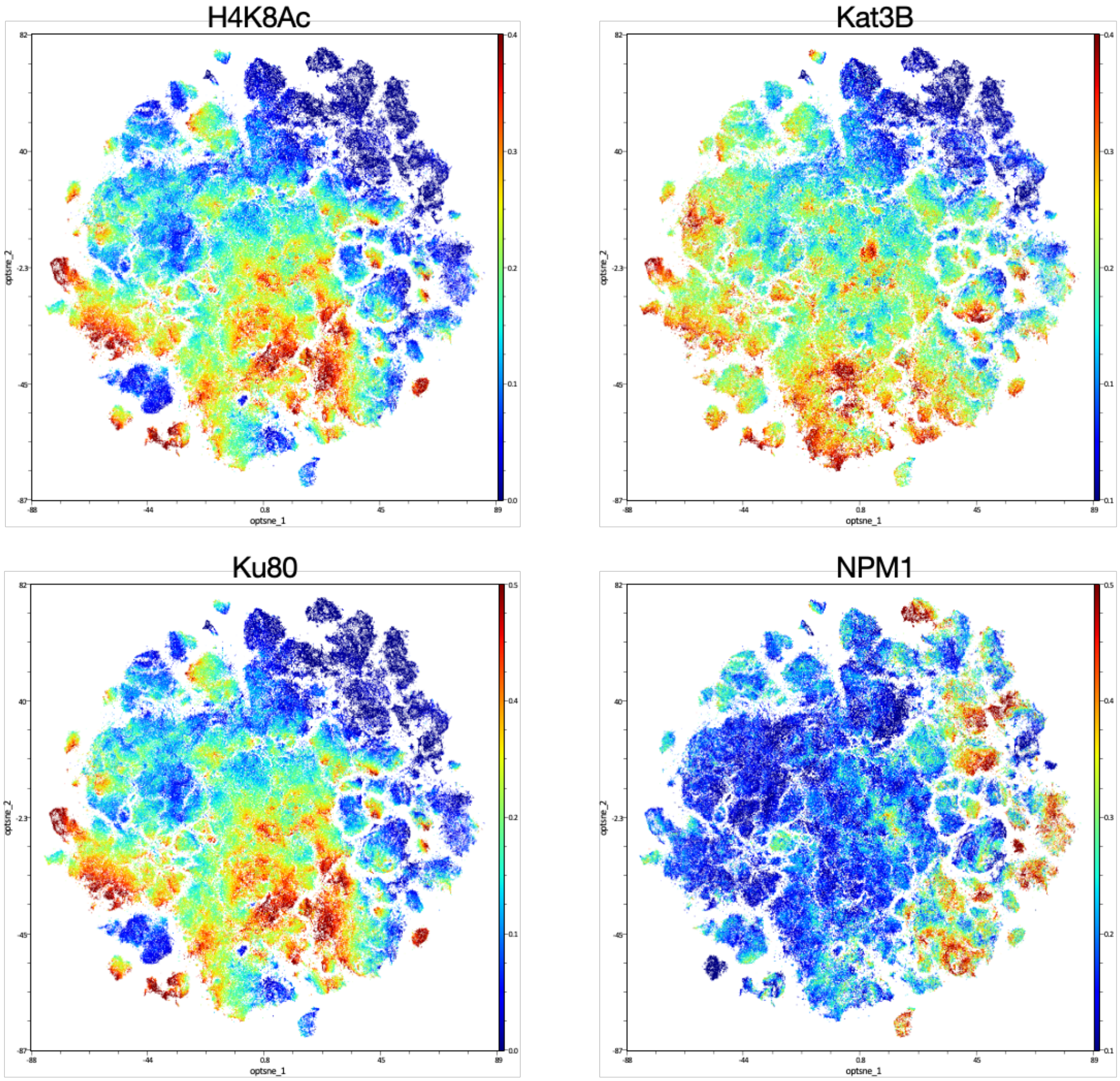

Figure S9. Single cell protein expression distribution in Optsne plots.

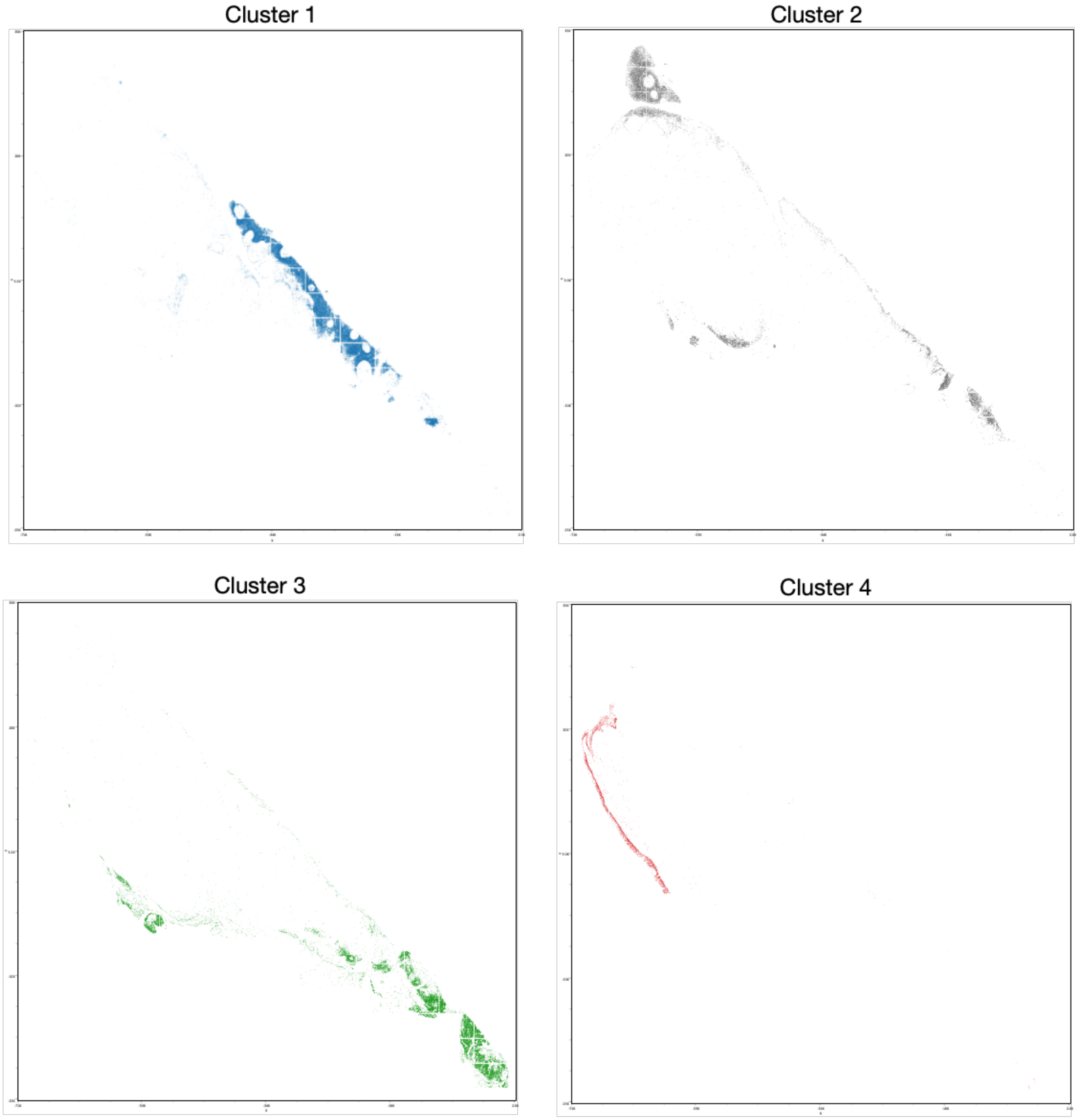
Cluster 1
Cluster 2
Cluster 3
Cluster 4

Cluster 5

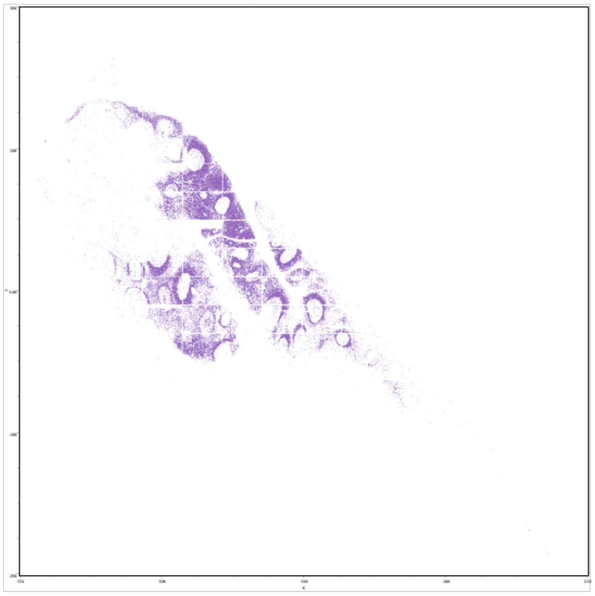

Cluster 6

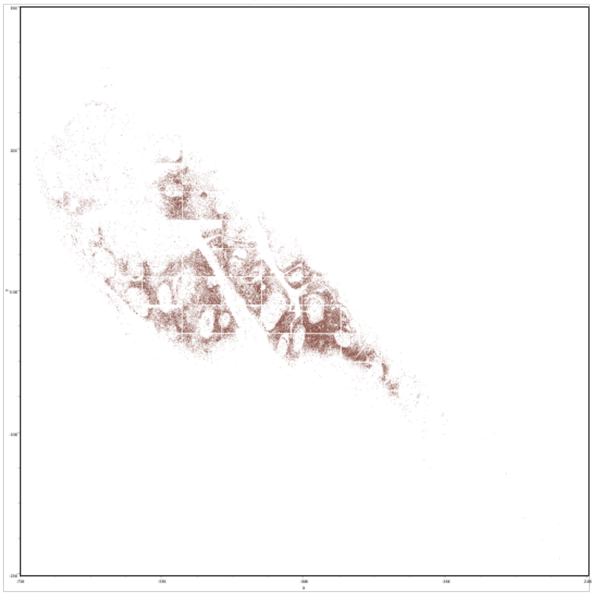

Cluster 7

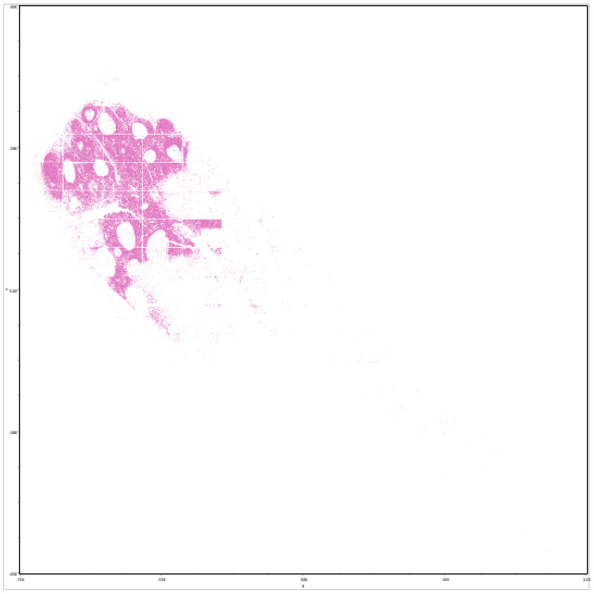

Cluster 8

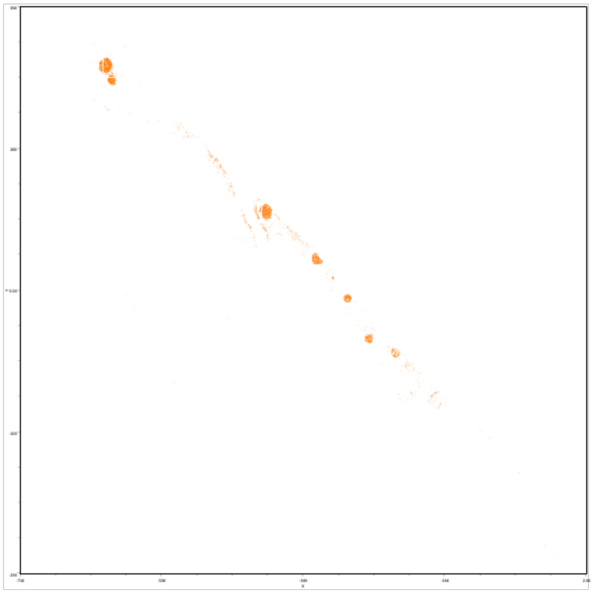

Cluster 9

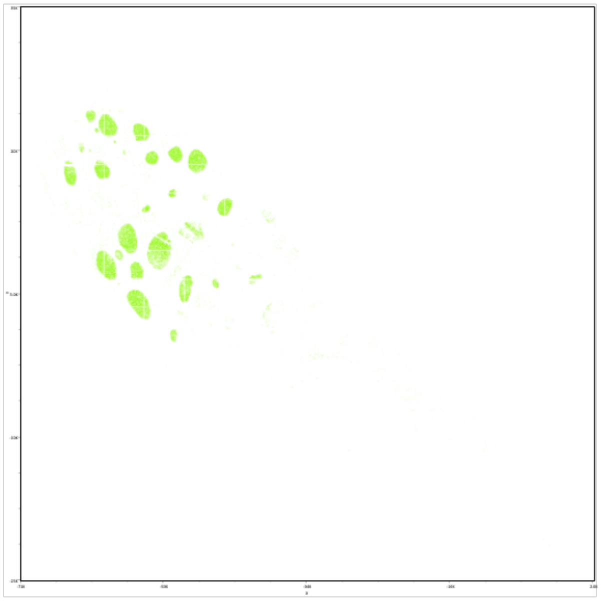

Cluster 10

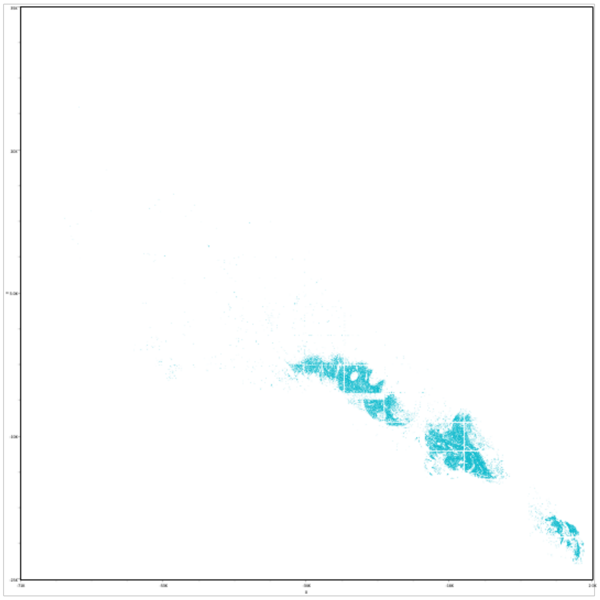

Cluster 11

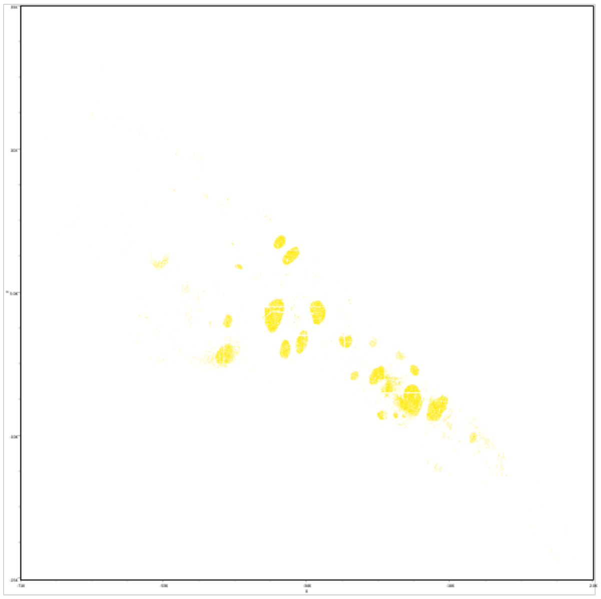

Cluster 12

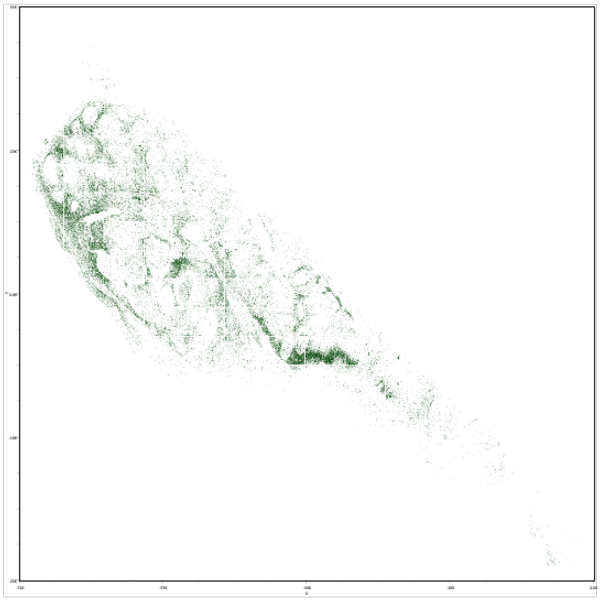

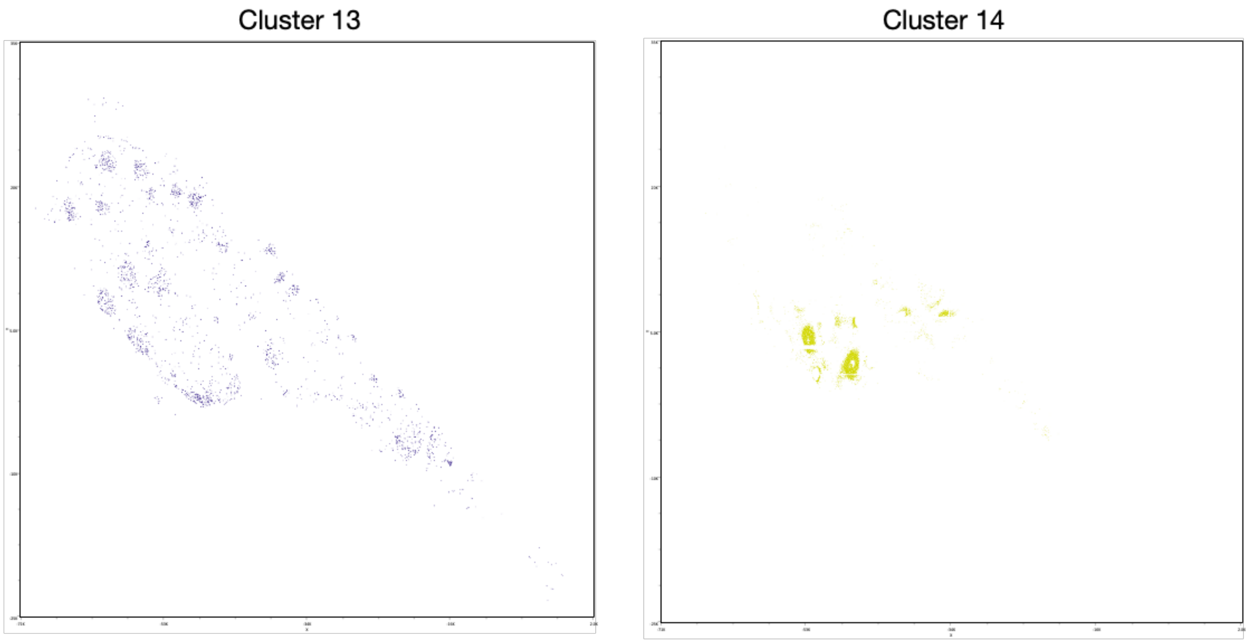

Figure S10. Anatomical locations of the individual cells from different cell clusters.
